# Combined utility of Ki-67 index and tumor grade to stratify patients with pancreatic ductal adenocarcinoma who underwent upfront surgery

**DOI:** 10.1186/s12893-023-02256-4

**Published:** 2023-12-08

**Authors:** Bo Li, Xiaoyi Yin, Xiuwen Ding, Guoxiao Zhang, Hui Jiang, Cuimin Chen, Shiwei Guo, Gang Jin

**Affiliations:** 1 Department of Hepatobiliary Pancreatic Surgery, Changhai Hospital, Naval Medical University (Second Military Medical University), 168 Changhai Road, Shanghai, 200433 China; 2 Department of Hepatobiliary Pancreatic Surgery, Naval Medical Center of People’s Liberation Army, Naval Medical University (Second Military Medical University), 338 West Huaihai Road, Shanghai, 200052 China; 3 Clinical Research Center, Changhai Hospital, Naval Medical University (Second Military Medical University), 168 Changhai Road, Shanghai, 200433 China; 4 Department of Pathology, Changhai Hospital, Naval Medical University (Second Military Medical University), 168 Changhai Road, Shanghai, 200433 China

**Keywords:** Pancreatic ductal adenocarcinoma, Tumor grade, Ki-67, Patient stratification

## Abstract

**Objective:**

To investigate the prognostic prediction of a new indicator, combined by tumor grade and Ki-67, in patients with resected pancreatic ductal adenocarcinoma (PDAC).

**Methods:**

Data were retrospectively collected from consecutive patients who underwent primary resection of pancreas from December 2012 to December 2017. Tumor grade and Ki-67 were reviewed from routine pathological reports. G-Ki67 was classified as three categories as I (G1/2 and Ki-67 < 40%), II (G1/2 and Ki-67 ≥ 40%), and III(G3/4 and all Ki-67).

**Results:**

Cox regression analyses revealed that tumor stage (II vs. I: hazard ratio (HR), 3.781; 95% confidence index (CI), 2.844–5.025; *P* < 0.001; III vs. I: HR, 7.476; 95% CI, 5.481–10.20; *P* < 0.001) and G-Ki67 (II vs. I: HR, 1.299; 95% CI, 1.038–1.624; *P* = 0.022; III vs. I: HR, 1.942; 95% CI, 1.477–2.554; *P* < 0.001) were independent prognostic factors in the developing cohort. The result was rectified in the validation cohort. In subgroups analysis, G-Ki67 (II vs. I: HR, 1.866 ; 95% CI, 1.045–3.334; *P* = 0.035; III vs. I: HR, 2.333 ; 95% CI, 1.156–4.705; *P* = 0.018) also had a high differentiation for survival prediction.

**Conclusion:**

Our findings indicate that three-categories of G-Ki67 in resectable PDAC according to the routine pathological descriptions provided additional prognostic information complementary to the TNM staging system.

**Supplementary Information:**

The online version contains supplementary material available at 10.1186/s12893-023-02256-4.

## Introduction

Pancreatic ductal adenocarcinoma (PDAC) is the fourth leading cause of cancer-related death worldwide, with a 5-year survival rate of 9–10% [1]. Surgical resection following adequate adjuvant therapy offers a chance of cure.However, even with R0 resection margin, the 5-year survival rate is only nearly 20% [2, 3]. The current staging modalities for PDAC cannot identify patients with occult metastases and aggressive biology, which correlated with distant metastatic disease within the first year after operation [4, 5]. Therefore, a new indicator related with aggressive biology may facilitate prognostic stratification and precise therapy.

Ki-67 is a nuclear protein that has demonstrable utility as a prognostic marker for several malignancies [6–8], including PDAC [9, 10]. Ki-67 is present during all active phases of the cell cycle (G1, S, G2, and mitosis), but is absent from resting cells (G0). Importantly, expression of Ki-67 reflects tumor proliferation rates and correlates with initiation, progression, metastasis and prognosis of many tumors [11]. Although Ki-67 is broadly used as a proliferation marker, the physiologic function of Ki-67 still needs further exploration, so as to guiding the patients stratification.

Besides, previous research showed that Ki67 was associated with the grade of differentiation in hepatocellular carcinoma [12] and the high grade tumors were more likely allocated in the patients with high expression of Ki-67, so as in PDAC [10]. Whereas, neither Ki-67 nor tumor grade was efficiently to stratify patients with PDAC, due to the misclassification for Ki-67 [11] and the skewed distribution of tumor grade category, with moderate differentiation mainly [13]. Therefore, the combination of Ki-67 and tumor grade was used as a score for recurrence prediction in patients with breast cancer [14]. An indicator of combination of Ki-67 and tumor grade maybe facilitate for subgrouping of patients with PDAC.

Therefore, we investigated the expression patterns of Ki-67 and the tumor grade in resected PDAC by reviewing the pathological reports and analyzed the prognostic value of the combination of the two markers by three categories. We hypothesized that patients in early stage are more efficiently discriminated by the three-categoried of this new indicator. In addition to the TNM staging system, the new indicator could be a candidate marker to stratify patients into more specific risk groups. Hence, this study aimed to investigate the potential of an indicator combined with Ki-67 and tumor grade for prognostic prediction in patients with resected PDAC.

## Materials and methods

### Study population and data collection

A total of 1469 consecutive patients with a final pathological diagnosis of PDAC who underwent primary pancreatic resection in Changhai Hospital (Shanghai, China) during December 2012 to December 2017 were enrolled in this study. For all patients, the following demographic and clinicopathological variables were recorded in the database: sex, age, preoperative carbohydrate antigen 19 − 9 (CA19-9), tumor location (head/neck/uncinate, body/tail), R status (R1 or R0), tumor grade (G1/2/3/4), perineural invasion (PNI), lymphovascular invasion (LVI), Ki-67 index and adjuvant therapy. The staging was performed following the 8th edition American Joint Committee on Cancer (AJCC) [15]. Clinical and follow-up data were obtained from a prospective digital database. The inclusion criteria were patients who underwent surgery with curative intent and pathological records could be obtained. The exclusion criteria for this study were as follows: (1) patients with intraoperative metastasis (excluded lymph node metastases) or macroscopic evidence of margin involvement (R2); (2) patients who received neoadjuvant chemotherapy or radiotherapy; (3) patients with other malignancies in the past; (4) patients who died within 90 days after surgery; and (5) patients who were lost to follow up. Subsequently, 1182 patients were included; of these patients, 629 underwent operation from December 2012 to June 2015 composed the developing cohort, and 553 underwent operation from July 2015 to December 2017 composed the validation cohort. This study was approved by the Institutional Review Board of Changhai Hospital, informed consent was obtained from all subjects and/or their legal guardian(s).

### Manual counting of the Ki-67 index and definition of G-Ki67

The Ki-67 hot spot proliferation index was assessed manually by a pathologist with special interest in pancreatic pathology. The Ki-67 index was defined as the area with the highest number of Ki-67-positive cells out of 100 cells in the respective area. The best cut-off points with minimum *P*-value was found at a Ki-67 fraction of 40% and Ki-67 was classified as < 40% and ≥ 40%. Meanwhile, G1/2 and G3/4. Tumor grade was classified as G1/2 and G3/4. Therefore, there were four classification combined by two-categoried tumor grade and two-categoried Ki-67. Based on Kaplan–Meier curve for survival analysis, G-Ki67 was classified into three categories as I (G1/2 and Ki-67 < 40%), II (G1/2 and Ki-67 ≥ 40%), and III (G3/4 and any Ki-67).

### Follow-up protocol

The institutional follow-up was jointly completed by follow-up specialists, and third-party (LinkDoc Technology Co. Ltd. Beijing, China). The frequency of follow-ups was once per two-months during the first half-year after surgery, followed by once per half-year until 30th October, 2022, the cutoff date of follow-ups in this study. The follow-up methods included outpatient visits, contact by phone, mail, chatting software, or address. The follow-up endpoint overall survival (OS) was defined as the time from surgery to death. Patients who were still alive at the cutoff date of follow-ups were censored when they were last confirmed to be alive. We defined loss to follow-up as a no-show at the clinical follow-ups or the inability to contact patients or their family members by phone, mail, or address.

### Statistical analysis

Categorical data are presented as percentages. Distributional differences in baseline variables between the two cohorts were examined using the chi-squared test or Wilcoxon rank-sum test. Univariate and multivariate Cox regression analyses were performed to identify independent prognostic factors, and hazard ratios (HRs) were calculated. Variables with a *P* < 0.1 in univariate analyses were included in multivariate analyses using a forward selection algorithm. The Kaplan–Meier method and log-rank test were used to analyze “time to endpoints.” Analyses were performed using SPSS version 25.0 (IBM Corp., Armonk, NY, USA) and R. For all analyses, a two-tailed *P* < 0.05 was considered statistically significant.

## Results

### Study population

**Fig. 1:**
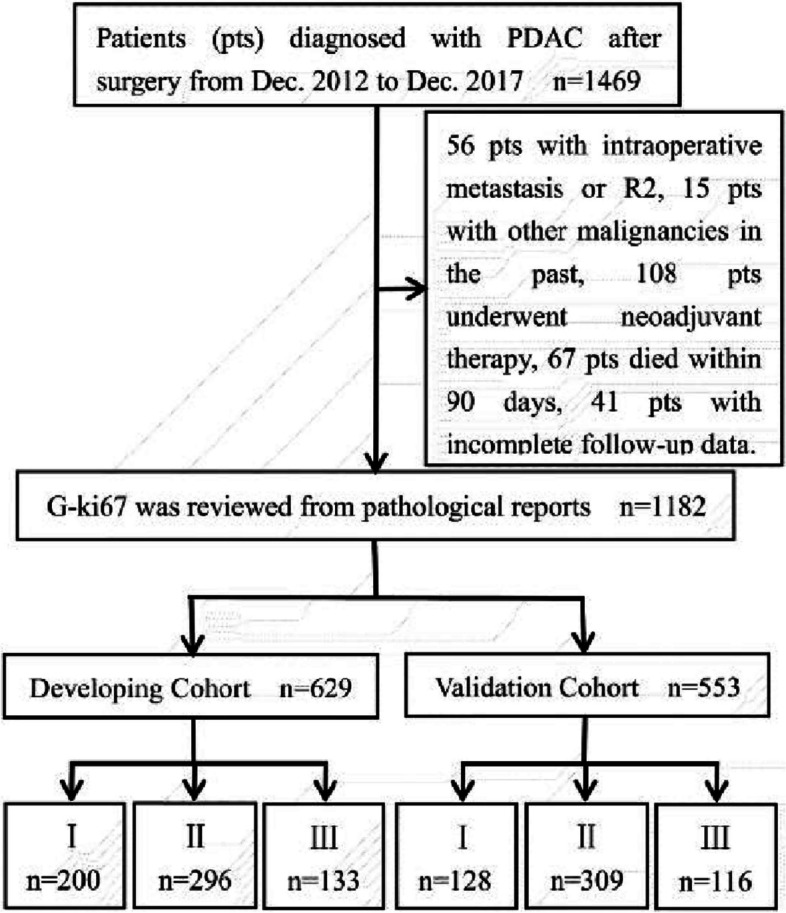
Flowchart depicting patient selection in the study

Of the 1469 consecutive patients in our study, 287 were excluded because they had intraoperative metastasis or R2 (*n* = 56), underwent neoadjuvant therapy (*n* = 108), had other malignancies in the past (*n* = 15), died within 90 days (*n* = 67), or were lost to follow-up (*n* = 41). All patients enrolled were of Asian descent. The developing cohort comprised 629 patients, whereas the validation cohort consisted of 553 patients. In the developing cohort, 200, 296, and 133 patients were classified as G-Ki67 I, II, and III, respectively, similar to the 128, 309, and 116 patients in the validation cohort (Fig. 1). Relevant baseline variables were showed in Table 1.

**Table 1:** Baseline characteristics of patients in the developing and validation cohorts

	Developing cohort	Validation cohort	P
**Total**	629	553	
**Age**, ≤ 65/>65 (years)	308/321	267/286	0.814
**Sex**, male/female	387/242	337/216	0.837
**Tumor location**, head/neck/uncinate, body/tail	388/241	355/198	0.373
**CA19-9**, < 37/≥37 U/mL	177/452	127/426	0.563
**T stage**, T1/2/3	129/338/162	114/307/132	0.743
** N stage**, N0/1/2	182/294/153	203/203/147	0.612
**TNM stage**, I/II/III	144/332/153	159/247/147	0.635
**PNI**, with/without	599/30	515/38	0.121
**LVI**, with/without	90/539	98/455	0.109
**R status**, R0/R1	503/126	411/142	0.597
**Grade**, G1/2/3/4	72/426/123/8	65/374/105/9	0.954
**Grade**, G1-2/3–4	496/133	437/116	0.944
**Ki-67**, < 40%/≥40%	230/399	157/396	0.002
**G-Ki67**, I/II/III	200/296/133	128/309/116	0.002
**Adjuvant therapy**, with/without	356/273	329/224	0.314

*Abbreviations:* *CA19-9 *Carbohydrate antigen 19 − 9, *TNM T*umor–node–metastasis, *PNI *Perineural invasion, *LVI *Lymphovascular invasion

### The selection of best candidate for prognostic prediction among Ki-67, tumor grade and G-Ki67

On univariate analysis for the whole cohort including both the developing and validation cohorts, Ki-67 as a continuous variable showed a statistically significant negative association with survival (Table 2). The best cut-off points with minimum *P*-value was found at a Ki-67 fraction of 40%, followed by 30% and 20% (Table 2). And the baseline characteristics of patients in Ki-67 subgroups by the fraction of 40% was shown in Supplementary Table 1.

**Table 2:** Univariate analysis carried out with different Ki67 fraction cut-off points to find those best dividing the patients according to survival

Ki67 cut-off point	n	HR (95% CI)	*P*
Continuous variable	1182	1.006 (1.002–1.010)	**0.005**
≥ 20% vs. <20%	1076 v.s.106	1.430 (1.090–1.876)	**0.010**
≥ 30% vs. <30%	980 vs. 202	1.359 (1.112–1.661)	**0.003**
≥ 40% vs. <40%	795 vs. 387	1.296 (1.105–1.520)	**0.001**
≥ 50% vs. <50%	546 vs. 636	1.062 (0.914–1.233)	0.433
≥ 60% vs. <60%	339 vs. 843	1.109 (0.936–1.313)	0.234
≥ 70% vs. <70%	140 v.s.1042	1.223 (0.957–1.562)	0.107

*Abbreviations:* *CI *Confidence interval, *HR *Hazard ratio

**Fig. 2:**
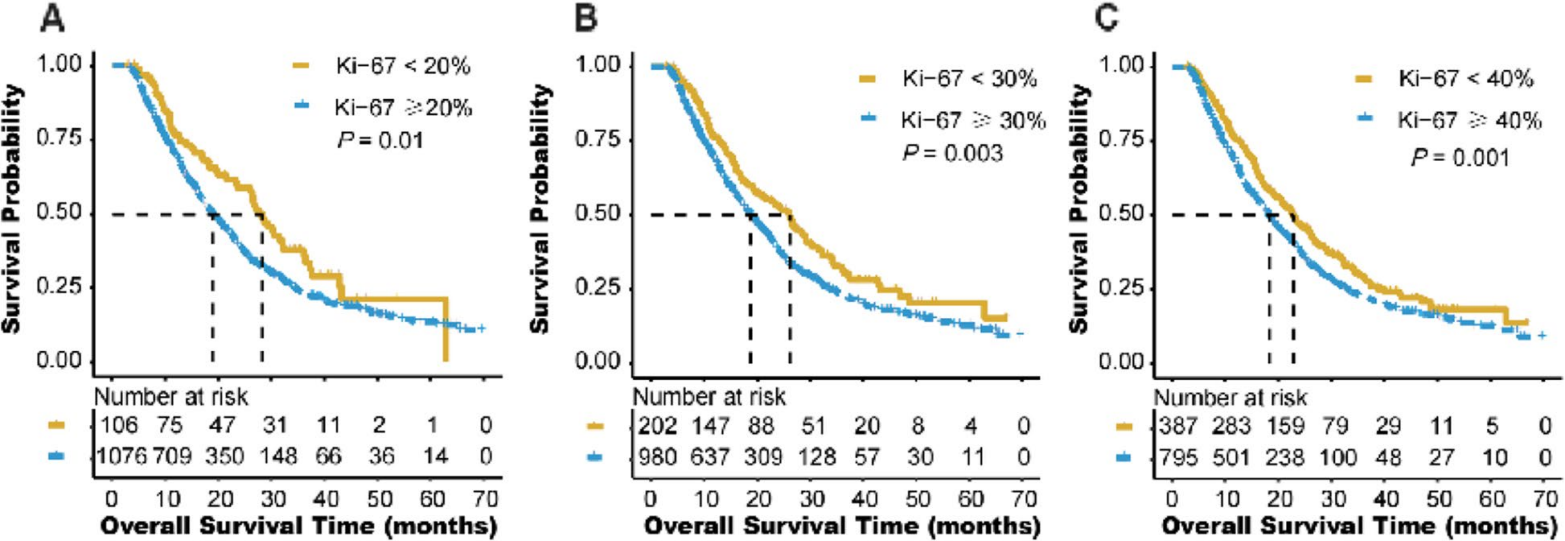
Kaplan-Meier diagrams showing over survival for Ki-67 index with cut-off points as 20% (**A**), 30% (**B**), and 40% (**C**). *P*-values for the log-rank test are shown in each panel

**Fig. 3:**
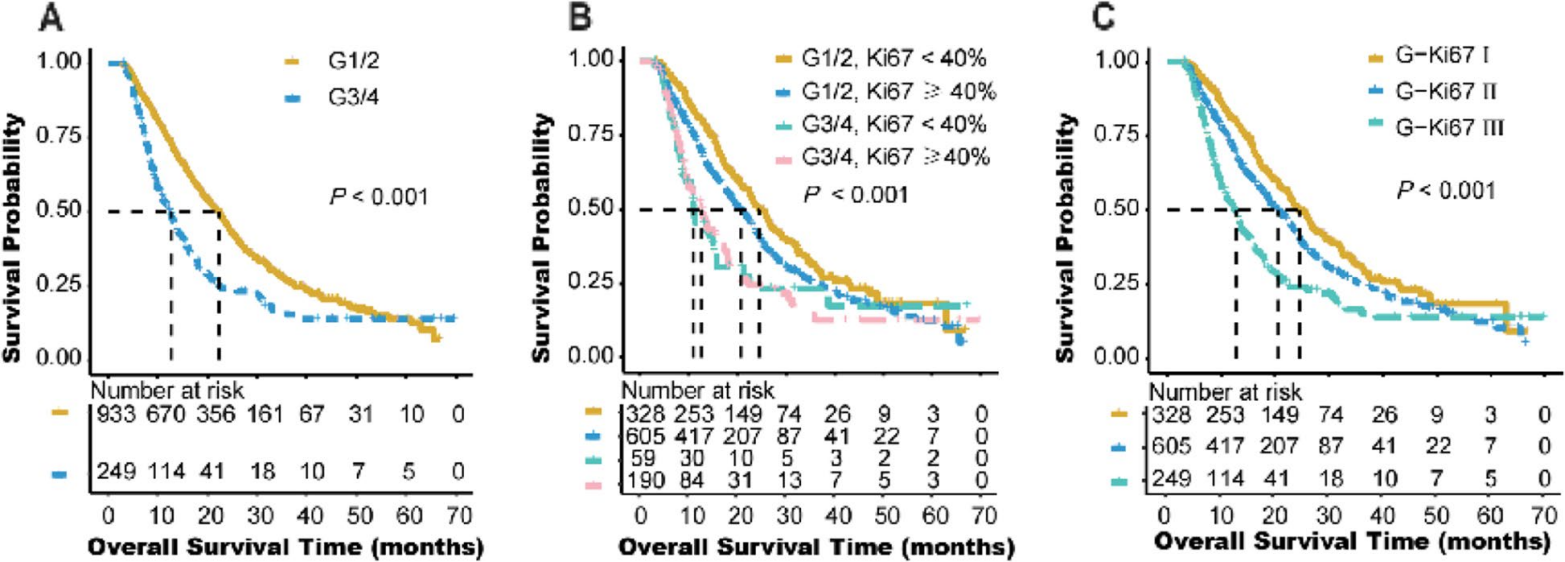
Kaplan-Meier diagrams showing over survival for tumor grade with 2 categories (**A**), G-Ki67 with 4 categories (**B**), and G-Ki67 with 3 categories (**C**). *P*-values for the log-rank test are shown in each panel

Survival curves for Ki-67 with cut-off points of 20%, 30% and 40% are shown in Fig. 2. Patients with G3/4 had a worse prognosis than G1/2 (HR, 1.785; 95%CI 1.493–2.134, *P* < 0.001, Fig. 3A). Whereas, due to the classification imbalance for tumor grade and Ki-67, neither of the two markers could efficiently classify patients for prognosis alone. By combination of Ki-67 and tumor grade as G-Ki67, we classified tumor into 4 categories as G1/2 and Ki-67 < 40%, G1/2 and Ki-67 ≥ 40%, G3/4 and Ki-67 < 40%, and G3/4 and Ki-67 ≥ 40% and the survival curve is shown in Fig. 3B. And because the curves for G3/4 and Ki-67 < 40%, and G3/4 and Ki-67 ≥ 40% were quite difficult to distinguish from each other, those 2 categories were merged as one category. Therefore, G-Ki67 could divided patients with PDAC into 3 subgroups as I (G1/2 and Ki-67 < 40%), II (G1/2 and Ki-67 ≥ 40%), and III (G3/4 and all Ki-67) in Fig. 3C.

### G-Ki67 was an independent prognostic indicator for PDAC

**Fig. 4:**
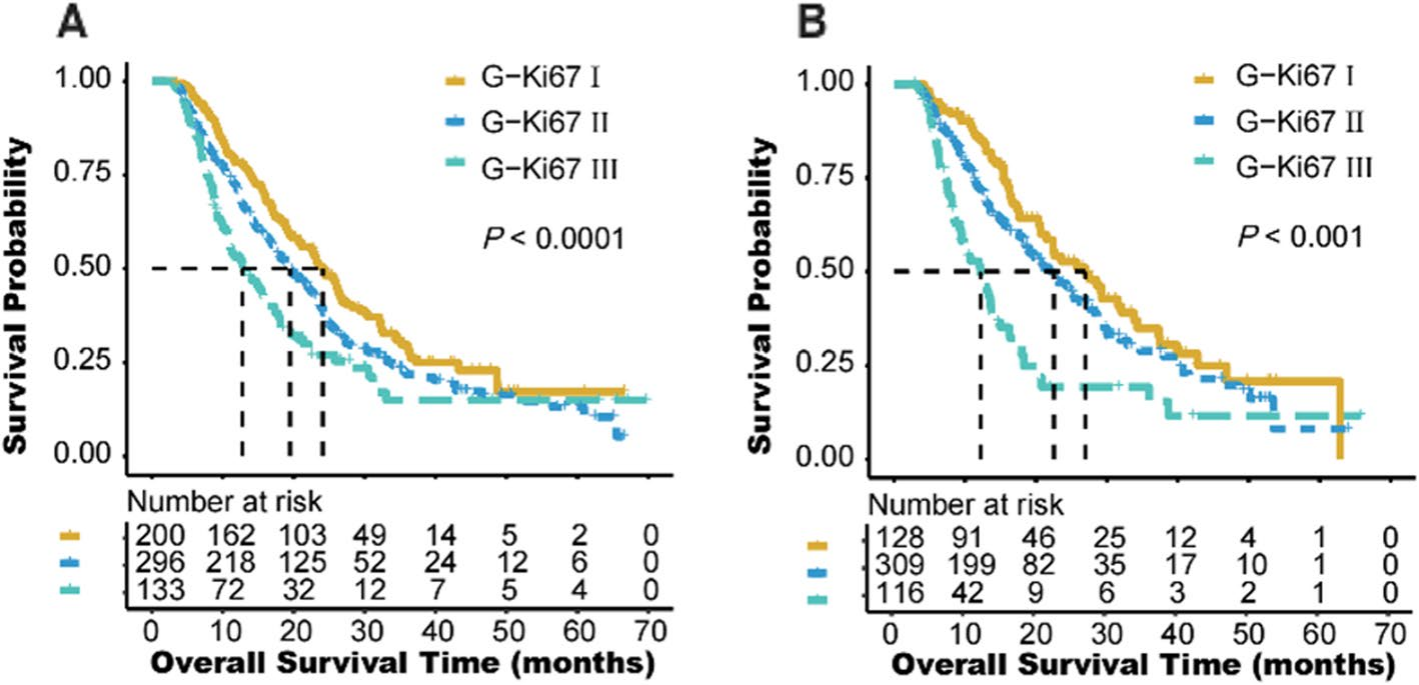
Kaplan-Meier diagrams showing over survival for G-Ki67 in the developing cohort (**A**) and validation cohort (**B**). *P*-values for the log-rank test are shown in each panel

In further, we performed a Cox regression analysis to examine the effect of postoperative clinicopathological parameters on prognosis. Univariate analyses revealed that T stage, N stage, TNM stage, adjuvant therapy, tumor grade (G3/4 vs. G1/2: HR, 1.551; 95% CI, 1.227–1.960; *P* < 0.001), Ki67 (≥ 40% vs. <40%: HR, 1.327; 95% CI, 1.085–1.622; *P* = 0.006), and G-Ki67 (II vs. I: HR, 1.308; 95% CI, 1.047–1.633; *P* = 0.018; III vs. I: HR, 1.821; 95% CI, 1.386–2.391; *P* < 0.001; Fig. 4) were significantly associated with OS in the developing cohort (Table 3). Except for Ki67 (≥ 40% vs. <40%: HR, 1.266; 95% CI, 0.972–1.647; *P* = 0.08) and R status (R1 vs. R0: HR, 1.381; 95% CI, 1.074–1.774; *P* = 0.012), the analysis results of the validation cohort were similar to those of the developing cohort (Table 3). Furthermore, the multivariate analysis confirmed that TNM stage (II vs. I: HR, 3.781; 95% CI, 2.844–5.025; *P* < 0.001; III vs. I: HR, 7.476; 95% CI, 5.481–10.20; *P* < 0.001) ,and G-Ki67 (II vs. I: HR, 1.299; 95% CI, 1.038–1.624; *P* = 0.022; III vs. I: HR, 1.942; 95% CI, 1.477–2.554; *P* < 0.001) and adjuvant therapy (without vs. with: HR, 2.788; 95% CI, 2.275–3.416; *P* < 0.001) (Table 4). The abovementioned independent prognostic factors were also validated in the validation cohort (Table 4).

**Table 3:** Univariate Cox regression analyses of clinicopathological features associated with OS of patients with PDAC

	Developing cohort		Validation cohort	
	HR (95% CI)	*P*	HR (95% CI)	*P*
**T stage**		**0.003**		**0.011**
T2 vs. T1	1.091 (0.853–1.396)	0.488	1.566 (1.142–2.145)	**0.005**
T3 vs. T1	1.533 (1.159–2.028)	**0.003**	1.631 (1.136–2.342)	**0.008**
** N stage**		**< 0.001**		**< 0.001**
N1 vs. N0	4.699 (3.593–6.146)	**< 0.001**	3.515(2.533–4.878)	**< 0.001**
N2 vs. N0	7.478 (5.576–10.03)	**< 0.001**	5.789 (4.022–8.332)	**< 0.001**
**TNM stage**		**< 0.001**		**< 0.001**
TNM II vs. TNM I	3.822 (2.879–5.072)	**< 0.001**	3.315 (2.304–4.767)	**< 0.001**
TNM III vs. TNM I	7.234 (5.310–9.856)	**< 0.001**	6.158 (4.131–9.179)	**< 0.001**
**Grade**, G3/4 vs. G1/2	1.551 (1.227–1.960)	**< 0.001**	2.220 (1.683–2.929)	**< 0.001**
**PNI**, with vs. without	0.648 (0.413–1.017)	0.059	1.834 (1.084–3.103)	0.024
**LVI**, with vs. without	1.693 (1.289–2.223)	**< 0.001**	1.836 (1.320–2.555)	**< 0.001**
**Ki67**, ≥ 40% vs. <40%	1.327(1.085–1.622)	**0.006**	1.266(0.972–1.647)	0.080
**G-Ki67**		**< 0.001**		**< 0.001**
II vs. I	1.308 (1.047–1.633)	0.018	1.316 (0.973–1.780)	0.075
III vs. I	1.821 (1.386–2.391)	**< 0.001**	2.688 (1.889–3.826)	**< 0.001**
**R status**, R1 vs. R0	1.050 (0.830–1.329)	0.684	1.381 (1.074–1.774)	**0.012**
**Adjuvant therapy**, with vs. without	1.675 (1.520–1.845)	**< 0.001**	1.505 (1.318–1.718)	**< 0.001**

*Abbreviations:* *CI *Confidence interval, *OR *Odds ratio, *TNM *Tumor–node–metastasis

**Table 4:** Multivariate Cox regression analyses of clinicopathological features associated with OS of patients with PDAC

	Developing cohort		Validation cohort	
	**HR (95% CI)**	***P***	**HR (95% CI)**	***P***
**TNM stage**		**< 0.001**		**< 0.001**
II vs. I	3.781 (2.844–5.025)	**< 0.001**	3.580 (2.481–5.165)	**< 0.001**
III vs. I	7.476 (5.481–10.20)	**< 0.001**	5.928 (3.968–8.857)	**< 0.001**
**G-ki67**		**< 0.001**		**< 0.001**
II vs. I	1.299 (1.038–1.624)	**0.022**	1.374(1.011–1.867)	**0.042**
III vs. I	1.942 (1.477–2.554)	**< 0.001**	2.756 (1.920–3.956)	**< 0.001**
**Adjuvant therapy**, without vs. with	2.788 (2.275–3.416)	**< 0.001**	2.232 (1.703–2.924)	**< 0.001**

*Abbreviations:* *CI *Confidence interval, *HR *Hazard ratio, *TNM *Tumor–node–metastasis

### G-Ki67 showed valuable prognosis prediction in subgroups of PDAC

**Fig. 5:**
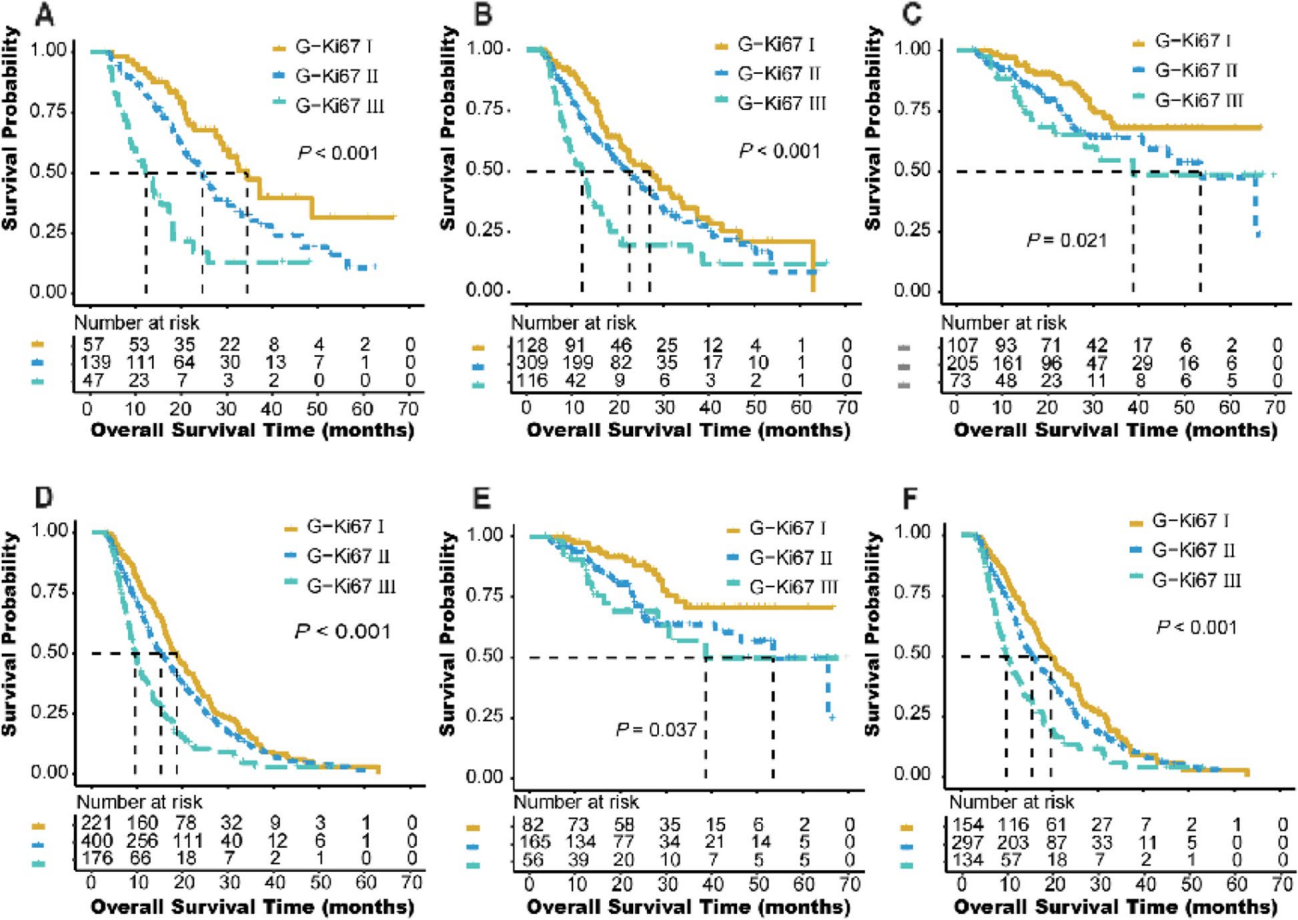
Kaplan-Meier diagrams showing over survival for G-Ki67 with 3 categories in subgroups for T1 (**A**), T2 (**B**), N0 (**C**), N1/2 (**D**), stage I (**E**), and stage T1/2N1/2 (**F**). *P*-values for the log-rank test are shown in each panel

Moreover, compared with G-Ki67 I, G-Ki67 III (G3/4) had a significantly worse prognosis in subgroups of patients with T stage T1, T2, T3, N stage N0 and N1/2, tumor stages I, and stage T1/2N1/2 (*P* < 0.05, Table 5, Fig. 5). However, compared with G-Ki67 I, G-Ki67 II had a significantly worse outcome in subgroups of patients with T stage T1, T2, N stage N0 and N1/2, and tumor stages I (*P*<0.05), not in stage T1/2N1/2 (*P*=0.051, Table 5).

**Table 5:** Overall survival analysis for G-Ki67 in subgroups

	n	G-Ki67, II vs. I		G-Ki67, III vs. I	
		HR (95% CI)	*P*	HR (95% CI)	*P*
**T1**	243	1.896 (1.193–3.011)	**0.007**	4.841 (2.819–8.314)	**< 0.001**
**T2**	645	1.299 (1.011–1.670)	**0.041**	1.714 (1.273–2.310)	**< 0.001**
**T3**	294	0.959 (0.682–1.350)	0.811	2.223 (1.451–3.406)	**< 0.001**
**N0**	385	1.694 (1.030–2.788)	**0.038**	2.274 (1.241–4.168)	**0.008**
**N1/2**	797	1.247 (1.030–1.510)	**0.024**	2.267 (1.801–2.855)	**< 0.001**
**T1/2N0 (Stage** I)	303	1.866 (1.045–3.334)	**0.035**	2.333 (1.156–4.705)	**0.018**
**T1/2N1/2**	585	1.252 (0.999–1.568)	0.051	2.130 (1.629–2.785)	**< 0.001**

*Abbreviations:* *CI *Confidence interval, *HR H*azard ratio, *TNM T*umor–node–metastasis

## Discussion

Based on two representative, well-characterized cohorts of 1182 patients with sporadic PDAC, we newly showed in our study that the three-categoried G-Ki67 assessed by routine pathological examination was a reliable prognostic indicator for patients with PDAC, especially for subgroups in early stage, such as T1 and TNM stage I.

The Ki-67 index provides an objective measurement to quantify the proliferation index and is used in numerous tumors for prognosis prediction [16–19]. In pancreatic tumors, Ki-67 has been used as a indicator for classification of pancreatic endocrine neoplasms grade [20], and Ki-67 > 5% predicted worse recurrence free survival [21]. Moreover, in pancreatic cancer, previous researches indicated that Ki-67 was an independent predictive factor for postoperative recurrence within one year [22], and also was a promising marker for the prediction of overall survival [23, 24]. Interestingly, Ki-67 combined with other markers [25] and/or clinicopathological predictors [10], may assist to better predict survival in resected pancreatic cancer, which is like as previous reported markers, such as glandular pattern [26], preoperative Ca19-9 levels [27], cancer-cell-derived sialylated IgG [28], matrix metalloproteinase 7 [29], and node-positive disease [30, 31]. However, a review summarized that several researches found no association between Ki-67 and survival in pancreatic cancer [32]. The inconsistency of the above-mentioned relationship of Ki-67 and survival for pancreatic cancer may contributed to the difference in sample size, cut-off value of Ki-67 [10, 23–25] and lack of reproducibility for Ki-67 [33]. Therefore, it is necessary to explore a method to facilitate the utilization of prognostic prediction for Ki-67 in clinical practice. Moreover, previous study suggested Ki-67 index correlated strongly with tumour grade in PDAC [10]. Hence the combination of Ki-67 and tumor grade, a routine pathological element, may optimized the efficiency of prognostic prediction, which was also used in previous studies in PDAC [10] and breast cancer [14].

Due to the lack of a standard scoring method for Ki-67 [34], we attempted to find the best cut-off value of Ki-67 for survival analysis with a large cohort, so as to avoid the bias form misclassification of Ki-67 [34]. In the current study, we found cut-off value as 40% of Ki-67, the same as previous report [25], could stratify the whole cohort of patients into two groups with significantly different outcomes. However, Ki-67 was not an independent factor for outcome prediction. In further, the three-categories of G-Ki67 could successfully separate patients into three groups based on survival outcome and G-Ki67 was also independently associated with prognosis in both developing and validation cohorts. And it indicated that G-ki67 was superior to Ki-67 or tumor grade for patients stratification in PDAC. Moreover, in early-stage patients (such as stage I), the survival outcome of G-Ki67 II and G-Ki67 III were both poor, that those patients may benefit from intensive surveillance after surgery, so as to optimize the disease management [35]. Considering the biological effects of Ki-67 and tumor grade, G-Ki67 may not only be a new indicator for patients stratification after surgery, but also be a candidate marker for response evaluation for adjuvant therapy [36, 37] or neoadjuvant therapy with the help of Ki-67 index evaluation by biopsy tissue [38]. Meanwhile, previous research suggested that Ki-67 was positively correlated with microvascular density, standing for angiogenesis, which provides a biological basis for the potential use of novel combinations of angiogenesis inhibitors and anti-proliferative chemotherapeutic drugs in the treatment of PDAC [39]. Therefore, G-Ki67 classification may provide useful information in clinical decision-making for precision management of PDAC.

Our study has important strengths. Firstly, our study demonstrates that the three-categoried indicator based on Ki-67 index and tumor grade is robust in prognostic assessment that outperforms the two-tiered Ki-67 index and two-tiered tumor grade in resected PDAC. Secondly, the three-categoried G-Ki67 has a good performance for outcome prediction in our two cohorts of previously untreated tumors from a high volume center. Thirdly, the novel classification system could be applicable in routine pathological descriptions of PDAC. Hence, the classification method is more likely to be used in other center for clinical practice and clinical trials. Finally, G-Ki67 is the representative for differentiation and proliferation of tumor cells, which were closely correlated with malignant behaviors, and the outcome prediction performance of G-Ki67 may also exist in other malignant tumors.

The present study also has several limitations that require consideration. Firstly, our study has the intrinsic shortcomings of any retrospective study. Secondly, the evaluation of Ki-67 index by approaches of IHC is lack of reproducibility, and the digital image analysis could be used for the precise quantification [40], but which may compromise the convenience of the clinical routine practice. Finally, the external validation is ongoing.

## Conclusion

Our findings indicate that three-categoried G-Ki67 in resectable PDAC according to the routine pathological descriptions provided independent prognostic information complementary to the TNM staging system. Accurate prognostication can assist patient selection for intensive surveillance and personalized treatment regimens, especially for patients in the early stage but with worse prognosis.

### Supplementary Information

**Additional file 1: Supplementary Table 1.** Baseline characteristics of patients in Ki-67 subgroups.

## Data Availability

The data that support the findings of this study are available from the corresponding author upon reasonable request.
