# Supplementary material for: Combined utility of Ki-67 index and tumor grade to stratify patients with pancreatic ductal adenocarcinoma who underwent upfront surgery
Source: BMC Surg. 2023 Dec 8;23:370. doi: 10.1186/s12893-023-02256-4 (PMC10704770; doi:10.1186/s12893-023-02256-4)
Supplement: Supplementary file 1 — Additional file 1: Supplementary Table 1. Baseline characteristics of patients in Ki-67 subgroups. [file 12893_2023_2256_MOESM1_ESM.docx]

**Supplementary Table 1. Baseline characteristics of patients in Ki-67 subgroups.**

|  | **<40%** | **≥40%** | ***P*** |
| --- | --- | --- | --- |
| **Total** | 387 | 795 |  |
| **Age,** ≤65/>65 (years) | 184/203 | 391/404 | 0.597 |
| **Sex,** male/female | 227/160 | 497/298 | 0.201 |
| **Tumor location,** head/neck/uncinate, body/tail | 211/176 | 532/263 | <0.001 |
| **T stage,** T1/2/3 | 70/211/106 | 173/434/188 | 0.205 |
| **N stage,** N0/1/2 | 126/168/93 | 259/329/207 | 0.716 |
| **TNM stage,** Ⅰ/Ⅱ/Ⅲ | 100/194/93 | 203/385/207 | 0.400 |
| **PNI,** with/without | 363/24 | 751/44 | 0.644 |
| **LVI,** with/without | 51/336 | 137/658 | 0.074 |
| **R status,** R0/R1 | 317/70 | 597/198 | 0.257 |
| **Grade,** G1/2/3/4 | 45/283/55/4 | 92/517/173/13 | 0.012 |
| **Grade,** G1-2/3-4 | 327/60 | 606/189 | 0.001 |

Abbreviations: CA19-9, carbohydrate antigen 19-9; TNM, tumor–node–metastasis; PNI, perineural invasion; LVI, lymphovascular invasion.
